# Airway obstruction requiring tracheal intubation after fine-needle aspiration of the thyroid gland: a case report

**DOI:** 10.1186/s12893-022-01476-4

**Published:** 2022-01-28

**Authors:** Junko Kousaka, Tsuneo Imai, Masayuki Saito, Hirona Banno, Yukie Ito, Mirai Ido, Manami Goto, Takahito Ando, Yukako Mouri, Kimihito Fujii, Shogo Nakano

**Affiliations:** Division of Breast and Endocrine Surgery, Department of Surgery, Aichi Medical University, 1-1 Yazakokarimata, Nagakute, Aichi 480-1195 Japan

**Keywords:** Fine-needle aspiration, Airway obstruction, Tracheal intubation, Acute transient thyroid swelling, Nodular goiter, Adenomatous goiter, Aortic regurgitation, Thyroid gland

## Abstract

**Background:**

There have been no reports of tracheal intubation for airway obstruction after acute thyroid swelling following fine-needle aspiration (FNA) of the thyroid gland.

**Case presentation:**

A 58-year-old woman with a 22 mm × 13 mm right hypervascular thyroid nodule underwent FNA once with a 22G needle under ultrasonographic guidance. Shortly after the aspiration, ultrasound revealed hypoechoic swelling with a crack-like pattern. The patient was observed under bed rest in the Fowler position and received intravenous steroids. A computed tomography (CT) scan showed swelling not only of the thyroid but also of the retropharyngeal space, and the patient complained of difficulty swallowing saliva. Laryngeal fiberscopy revealed protrusion of the posterior pharyngeal wall, edematous changes in the mucosa of the pharynx and epiglottis, and retention of saliva. The patient was intubated awake and hydrocortisone was administered every 8 h. She was extubated 3 days after FNA and discharged without any complications.

**Conclusions:**

When neck swelling is noticed after FNA, ultrasonographic findings are especially important to assess potential causes. If airway obstruction is suspected, CT findings and fiberscope observation of the pharynx provide particularly useful information.

## Background

Fine needle aspiration (FNA) cytology is the procedure of choice in the diagnosis of thyroid nodules. The accuracy of FNA of the thyroid gland is extremely high, and the procedure is quite safe when performed under direct ultrasonographic (US) guidance. The extensive use of thyroid US guided FNA has determined a surprisingly increased detection of differentiated cancer and in about 15–30% of cases of “follicular neoplasm/suspicious for follicular neoplasm” lesions [1]. However, there have been a few reports of acute thyroid swelling after this procedure. In some cases, acute hematoma required surgical treatment [2–4], but more commonly, nonhemorrhagic transient swelling resolved without invasive intervention [5–8]. Herein, we report a case of acute thyroid swelling after FNA that resulted in airway obstruction requiring tracheal intubation.

## Case presentation

**Fig. 1:**
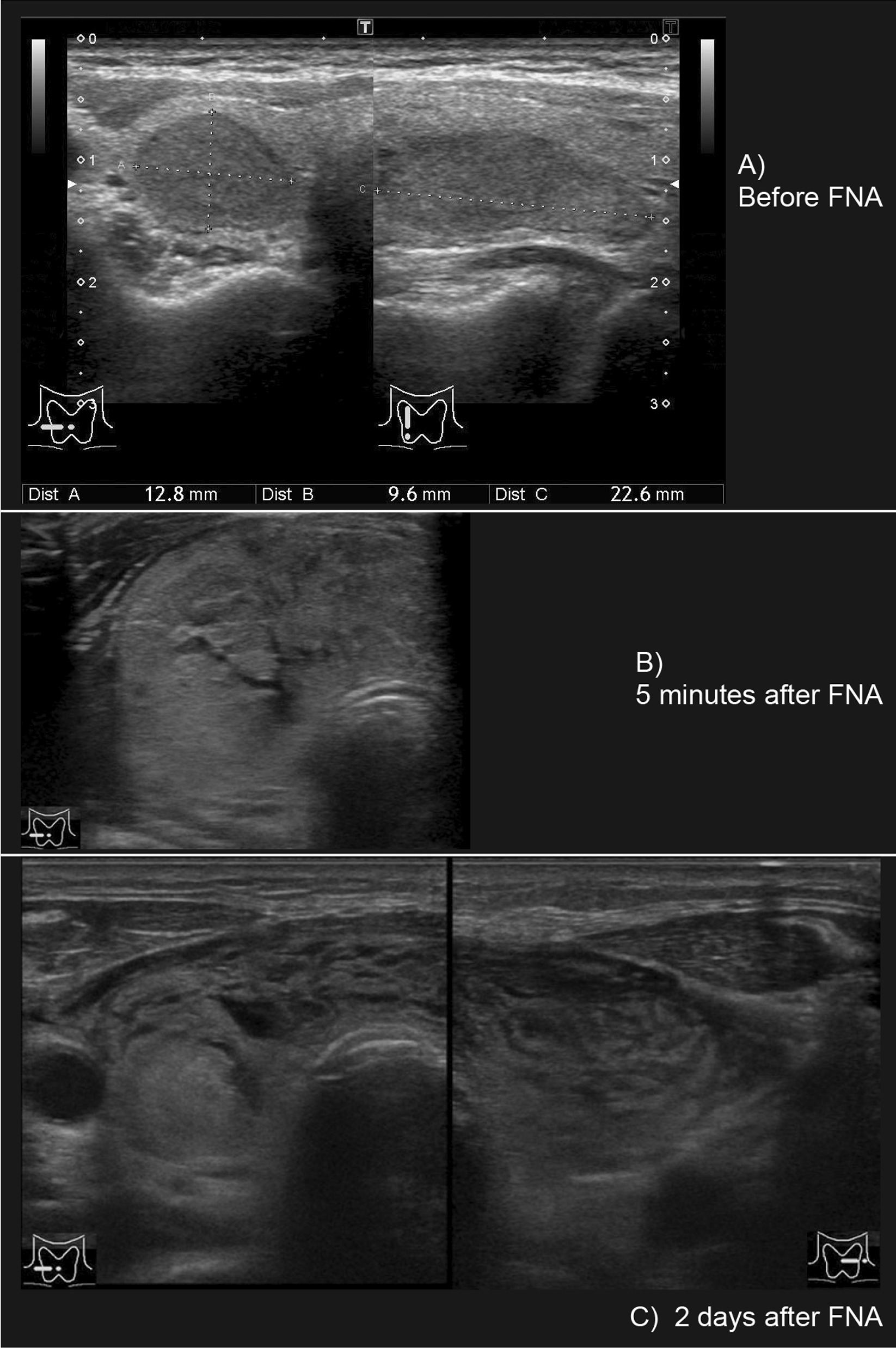
**A** US findings before FNA. An iso-hypoechoic mass (22.6 mm × 12.8 mm × 9.6 mm) is shown in the right lobe of thyroid gland. **B** US findings 5 min after FNA. The thyroid gland is massively enlarged; hypoechoic areas demonstrate a crack-like pattern, and there is fluid retention a few millimeters thick in the subcapsular area of the thyroid gland. **C** US findings 2 days after FNA. Swelling of the thyroid and surrounding tissues are rapidly decreased, although US findings of the thyroid gland still show a crack-like, hypoechoic pattern

**Fig. 2:**
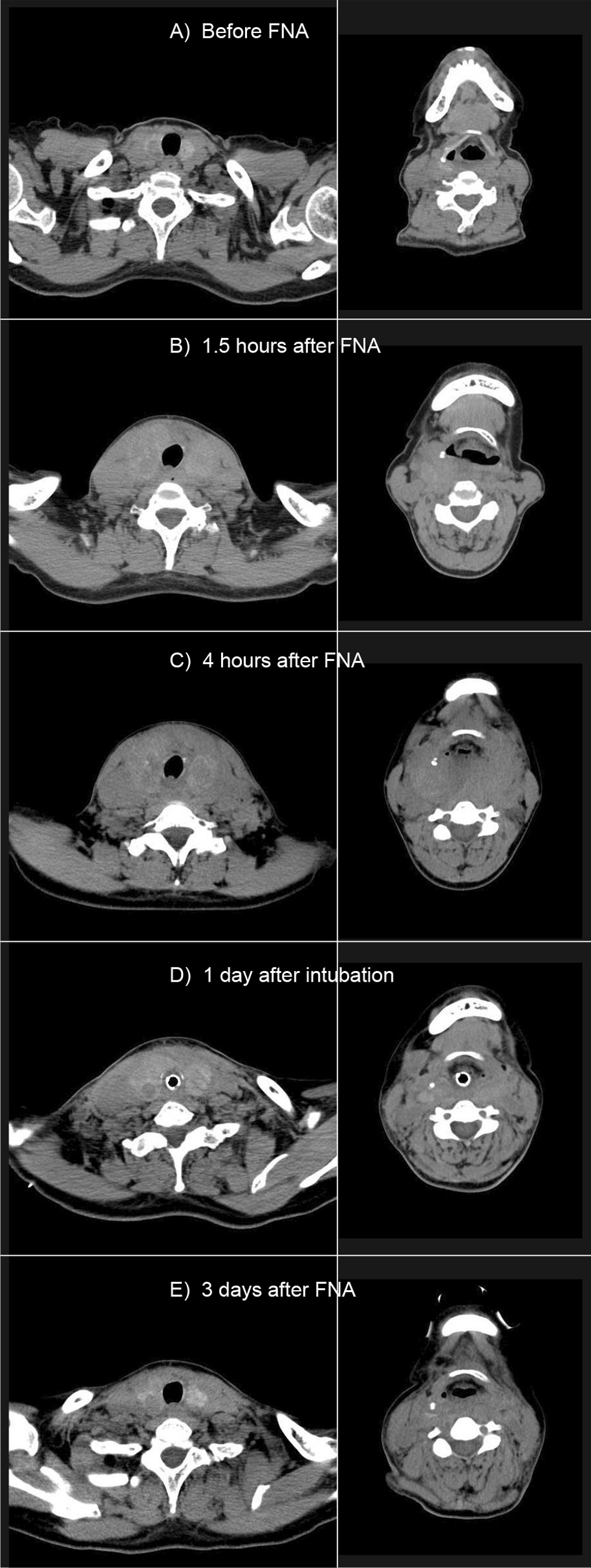
**A** CT findings before FNA. The thyroid nodule in the right lobe shows low signal intensity compared with surrounding thyroid gland tissue. The right internal jugular vein is dilated. The left panel shows a CT slide obtained at the thyroid gland level, while the right panel shows a slice at the hyoid bone level. **B** CT findings 1.5 h after FNA. There is swelling not only of the thyroid gland but also of the retropharyngeal, especially on the right side. The right internal jugular vein is dilated. **C** CT findings before intubation (4 h after FNA). Expansion of the retropharyngeal tissue has increased bilaterally, and the narrowness of the retropharyngeal space is evident. The right internal jugular vein is dilated. **D** CT findings 1 day after intubation. Swelling of the retropharyngeal tissue has subsided dramatically, and thyroid gland enlargement has decreased. The right internal jugular vein is still dilated. **E** CT findings 3 days after FNA. Swelling of the retropharyngeal tissue has resolved, and thyroid gland enlargement has further decreased. The right internal jugular vein was continuously dilated

A 58-year-old woman (height, 157 cm; weight, 53 kg; BMI, 1.51) was referred for outpatient FNA of the thyroid gland to evaluate a 22 mm × 13 mm right hypervascular thyroid nodule (Figs. 1A, 2A). She was taking prednisolone 2 mg and iguratimod 25 mg for rheumatoid arthritis and diuretics for continuing left-to-right shunt after patch repair of sinus of Valsalva aneurysm 15 years before. Her thyroid function was normal; free T3, 2.72 pg/ml; free T4, 1.23 ng/dl; TSH, 2.821 mIU/ml, and she had no known allergies. FNA was performed without anesthesia once using a 22G needle under US guidance. Shortly after aspiration, ultrasonography demonstrated hypoechoic swelling with a crack-like pattern as well as a small amount of fluid retention in the subcapsular space of the thyroid gland. Five minutes after the single needle pass, the patient complained of neck pain and difficulty swallowing saliva. She was observed under bed rest in the Fowler position. Neck swelling was evident (Fig. 1B), and hydrocortisone 100 mg was administered intravenously. A computed tomography (CT) scan performed 1.5 h after FNA showed swelling not only of the thyroid gland but also of the retropharyngeal space (Fig. 2B). The patient complained of dyspnea and she was transferred to the intensive care unit 3 h after FNA. Laryngeal fiberscopy revealed protrusion of the posterior pharyngeal wall, edematous changes in the mucosa of the pharynx and epiglottis, and retention of saliva. Her dyspnea worsened and stridor developed, although her SpO_2_ was 96%. A repeat CT scan showed increased swelling of the thyroid and surrounding areas, especially at the level of the hyoid bone. The swelling exhibited the same CT density as muscle, and it extended from the mid-pharyngeal level to the upper mediastinum (Fig. 2C). An endocrine surgeon, otolaryngologist, and intensive care physician discussed the patient’s ongoing management and decided on conservative observation rather than immediate surgery because the US findings showed diffuse swelling with a heterogeneous (crack-like) pattern (hypoechoic areas interspersed throughout the gland), which meant that the swelling was due to reversible edema and not hematoma. Furthermore, it was decided that the patient should be intubated early because progressive retropharyngeal swelling and edematous pharyngeal changes were expected to result in later difficulty with intubation. She was intubated awake and hydrocortisone 100 mg was administered every 8 h. US and CT assessment was performed every day. On the next day after intubation, swelling of the thyroid and surrounding tissues had rapidly decreased, although ultrasound still demonstrated the crack-like, hypoechoic appearance (Figs. 1C, 2D). CT of the thyroid gland showed findings similar to those seen before FNA, and the patient was extubated 3 days after FNA (Fig. 2E). Echocardiography revealed elevated central venous pressure due to continuing left-to-right shunt after patch repair of sinus of Valsalva aneurysm, in agreement with the dilation of the internal jugular vein observed throughout the hospital course. She was discharged 7 days after extubation without any complications. FNA findings of the thyroid gland were benign.

## Discussion and conclusions

The overall prevalence of acute, transient thyroid swelling was previously reported to be 0.46% [9], 0.15% [10], 0.13% [11], and 0.10% [6]. In past reports, this swelling subsided within 1–20 h, and none of the patients developed airway obstruction. This is the first report in the English literature to describe a patient who required tracheal intubation for airway obstruction after acute thyroid swelling following FNA. A fatal case of cervical hemorrhage was reported in a forensic journal, indicating the need to be aware of potentially fatal airway obstruction following FNA of the thyroid gland [12].

Acute, transient thyroid swelling exhibits several specific US findings. In the present case, characteristic hypoechoic “cracks” (i.e., a crack-like pattern) [13] were observed, along with swelling, in both lobes of the thyroid gland, despite the fact that only one lobe was punctured. These hypoechoic cracks reflect fluid accumulation in the loose interstitial space of the thyroid parenchyma. The acute nature of the swelling in this case and its spontaneous and relatively rapid resolution suggested diffuse capillary leakage caused by puncture of the thyroid parenchyma. In our case, relatively minimal hemorrhage may have coexisted with non-hemorrhagic swelling of the thyroid gland and surrounding tissues. The hypervascular nodule and elevated central venous pressure were risk factors for hemorrhage, but most of the swelling was probably non-hemorrhagic in nature because it resolved within 24–48 h after FNA.

The mechanism of acute, transient thyroid swelling remains unclear. Its acute onset and short recovery time suggest that intrathyroidal hemorrhage is an unlikely etiology. Other possible explanations include intrathyroidal edema induced by endogenous substances [14], or allergic reaction to metal needles, disinfectants, or ultrasound gel [15]. Acute transient thyroid swelling after catheterization of subclavian vein was reported in an apparently normal thyroid gland [16]. Fifteen minutes after multiple unsuccessful attempts to insert a central venous catheter in the neck, the authors noted progressive diffuse swelling of the neck. US findings revealed a diffusely swollen thyroid gland without evidence of bleeding. The swelling resolved spontaneously within 4 h, and the authors concluded that the normal thyroid gland was inadvertently punctured during the attempted catheterization. Thus, thyroid nodules themselves might not be inherently associated with a risk of acute, transient thyroid swelling.

Although it is rare, FNA can lead to acute, transient thyroid swelling. When neck swelling is noticed after FNA, US findings are especially important to assess potential causes. If airway obstruction is suspected, CT findings and fiberscope observation of the pharynx provide particularly useful information.

## Data Availability

Data sharing is not applicable to this article as no datasets were generated or analyzed during the current study.

## Abbreviations

FNA: Fine needle aspiration
US: Ultrasonographic
CT: Computed tomography
BMI: Body Mass Index
SpO_2_: Saturation pulse oxygen
